# Development and validation of a predictive model for febrile seizures

**DOI:** 10.1038/s41598-023-45911-9

**Published:** 2023-10-31

**Authors:** Anna Cheng, Qin Xiong, Jing Wang, Renjian Wang, Lei Shen, Guoqin Zhang, Yujuan Huang

**Affiliations:** Department of Emergency, Shanghai Children′s Hospital, School of Medicine, Shanghai Jiao Tong University, Shanghai, China

**Keywords:** Paediatric neurological disorders, Paediatric research

## Abstract

Febrile seizures (FS) are the most prevalent type of seizures in children. Existing predictive models for FS exhibit limited predictive ability. To build a better-performing predictive model, a retrospective analysis study was conducted on febrile children who visited the Children's Hospital of Shanghai from July 2020 to March 2021. These children were divided into training set (n = 1453), internal validation set (n = 623) and external validation set (n = 778). The variables included demographic data and complete blood counts (CBCs). The least absolute shrinkage and selection operator (LASSO) method was used to select the predictors of FS. Multivariate logistic regression analysis was used to develop a predictive model. The coefficients derived from the multivariate logistic regression were used to construct a nomogram that predicts the probability of FS. The calibration plot, area under the receiver operating characteristic curve (AUC), and decision curve analysis (DCA) were used to evaluate model performance. Results showed that the AUC of the predictive model in the training set was 0.884 (95% CI 0.861 to 0.908, p < 0.001) and C-statistic of the nomogram was 0.884. The AUC of internal validation set was 0.883 (95% CI 0.844 to 0.922, p < 0.001), and the AUC of external validation set was 0.858 (95% CI 0.820 to 0.896, p < 0.001). In conclusion, the FS predictive model constructed based on CBCs in this study exhibits good predictive ability and has clinical application value.

## Introduction

Febrile seizures (FS) are the most common type of convulsions in childhood, primarily affecting children between the ages of 6 months and 6 years^1^. FS often induce panic in parents, leading them to employ unnecessary or excessive management measures due to concerns about potential neurological damage, asphyxia, or even death during these episodes. Consequently, FS are common conditions in the pediatric emergency department^2^. The exact pathogenesis of FS is unknown, although some researchers suggest that it may be associated with factors such as infections, particularly viral infections, genetic susceptibility, and certain vaccinations^3,4^. While it is previously widely believed that most FS are harmless^5^, recent studies have revealed a correlation between FS and neurological, cognitive, and memory deficits^6–8^. Furthermore, research has demonstrated that individuals with FS are at an increased risk of developing epilepsy or psychiatric disorders compared to children without FS, with the risk escalating with each occurrence of FS^9,10^. Reports have also indicated an elevated risk of sudden death in patients with FS^11^. Therefore, further exploration of methods for predicting FS is still necessary.

For the prediction of FS, Cokyaman et al. conducted a study utilizing serum Brain-derived neurotrophic factor levels to predict FS, yielding the area under the curve (AUC) of 0.723^12^. Bakri et al. discovered that neurotrophin-3 predicted FS with an AUC of 0.678^13^. In Baek et al.'s study, hypomagnesemia was identified as an independent risk factor for FS, and when used to predict FS, it achieved an AUC of 0.731^14^. Liu et al.'s study employed neutrophil to lymphocyte ratio (NLR) and mean platelet volume (MPV)/platelet count (PLT) ratio (MPR) to predict FS, resulting in AUC values of 0.768 and 0.689, respectively^15^. On one hand, the existing FS predictive models demonstrated limited predictive ability, with AUC values below 0.8. On the other hand, complete blood counts (CBCs) have shown potential in predicting FS, such as the NLR single indicator with an AUC value of 0.768, which was close to 0.8^15^. By incorporating additional CBCs, the model's predictive ability could be significantly enhanced. Therefore, our study aims to establish an FS predictive model using CBCs that significantly improves predictive ability and holds practical value.

## Materials and methods

### Study design and patients

The study protocol was approved by the Ethics Committee of Children’s Hospital of Shanghai (approval number 2022R132). All methods were carried out in accordance with the relevant guidelines and regulations. Informed consent was obtained from the legal guardian(s) of each child.

**Figure 1:**
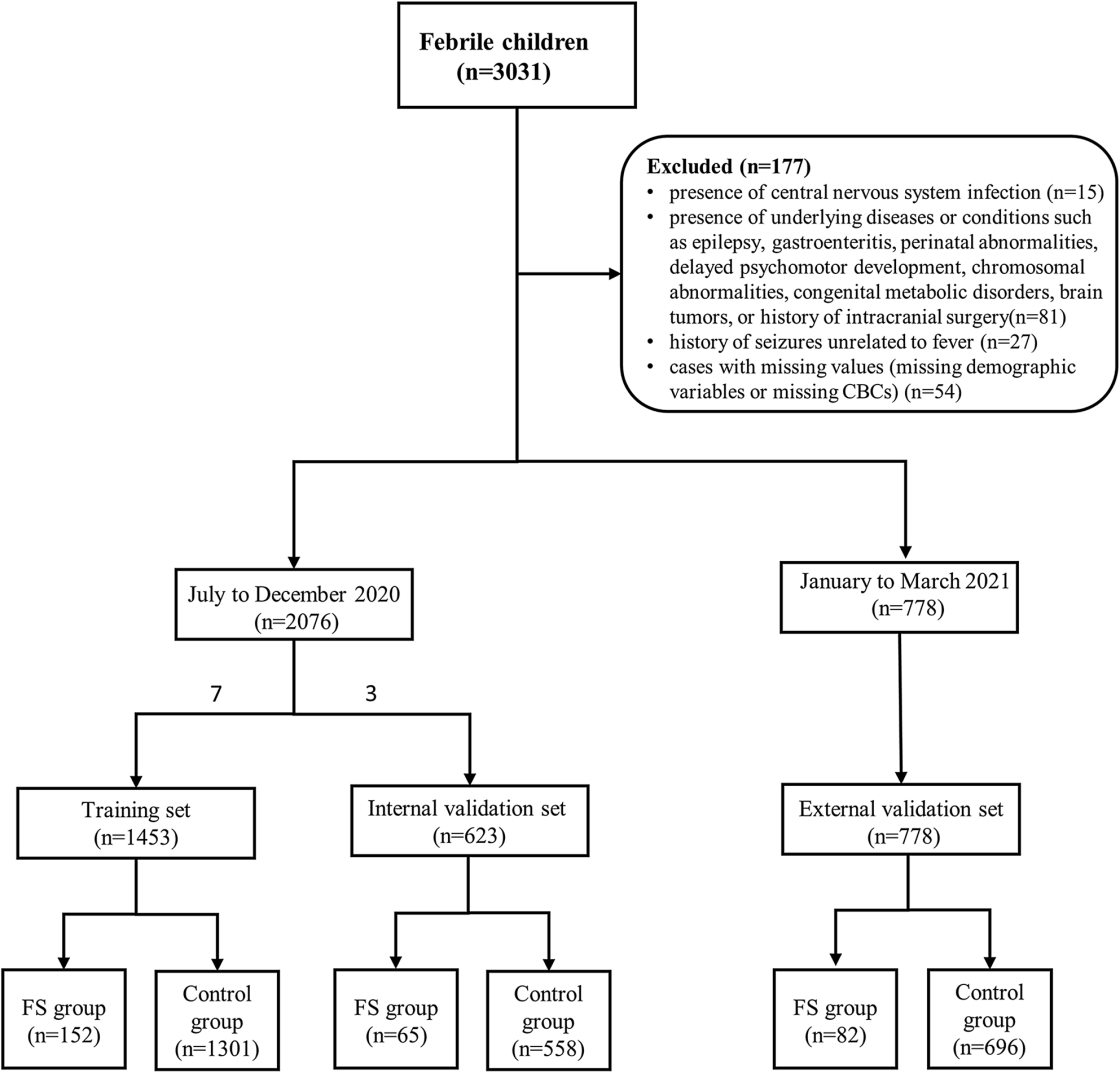
Flow diagram of participants selection.

We collected demographic data and CBCs of 3031 febrile children (328 children with FS and 2703 febrile children without seizures) who visited the emergency department of Children's Hospital of Shanghai from July 2020 to March 2021. All the children were aged between 6 months and 6 years old. The diagnosis of FS was performed according to the clinical practice guideline for the long-term management of the child with simple febrile seizures by the Subcommittee on FS American Academy of Pediatrics^16^. The exclusion criteria consisted of the following: (1) the presence of central nervous system infection; (2) the presence of underlying diseases or conditions such as epilepsy, gastroenteritis, perinatal abnormalities, delayed psychomotor development, chromosomal abnormalities, congenital metabolic disorders, brain tumors, or history of intracranial surgery; (3) history of seizures unrelated to fever; (4) cases with missing values (missing demographic variables or missing CBCs). Through screening, a total of 2854 children were included in the study, consisting of 299 children with FS and 2555 febrile children without seizures. The 2020 data was randomly divided into training set and internal validation set in a 7:3 ratio. 1453 children were included in the training set, and 623 children were included in the internal validation set. Data from January to March 2021 were used as the external validation set, with a sample size of 778. Data exclusion and data splitting were shown in Fig. 1.

### Variables

Demographic information, including age and gender, as well as CBCs were collected. The CBCs encompassed the following indicators: absolute neutrophil count (ANC), absolute lymphocyte count (ALC), absolute monocyte count (AMC), absolute eosinophil count (AEC), absolute basophil count (ABC), red blood cell count (RBC), hematocrit (Hct), mean corpuscular volume (MCV), hemoglobin (Hb), mean corpuscular hemoglobin (MCH), mean corpuscular hemoglobin concentration (MCHC), platelet count (PLT), mean platelet volume (MPV), red blood cell distribution width (RDW), platelet distribution width (PDW), and C-reactive protein (CRP). Additionally, the neutrophil to lymphocyte ratio (NLR) was calculated.

### Statistical analysis

Statistical analysis was performed using SPSS (version 25.0), STATA (version 17) and R language (version 4.2.3). Continuous variables were described using means and standard deviations (SD), while categorical variables were described using frequencies and percentages. The training set was utilized for model development, while the internal validation set, and external validation set was used for testing the model.

The least absolute shrinkage and selection operator (LASSO) method was used to select the predictors of FS. Variables with non-zero coefficients were selected in the LASSO regression model. Multivariate logistic regression model was used to predict FS. The coefficients obtained from the multivariate logistic regression were used to create a nomogram that predicts the probability of FS.

The receiver operating characteristic curve (ROC) was utilized to evaluate the predictive performance. The AUC was calculated for three sets to assess the model's discriminatory capacity. The discriminatory capacity was categorized as poor (AUC: 0.6–0.69), adequate (AUC: 0.7–0.79), good (AUC: 0.8–0.89), or excellent (AUC: 0.9–1.0)^17^. The validation of the nomogram involved assessing its discrimination (C-statistic) and calibration (calibration plot). Generally, a C-statistic value greater than 0.75 was considered indicative of relatively good discrimination.

Finally, we measured the applicability of the nomogram to clinical practice through decision curve analysis (DCA) and clinical impact curve (CIC). A significance level of p < 0.05 was considered statistically significant in all analyses.

The reporting of this study followed the guidelines of Transparent Reporting of a multivariable prediction model for Individual Prognosis Or Diagnosis (TRIPOD)^18^.

## Results

### Analysis of clinical profiles and laboratory variables in the training set

Within the training set, patients were divided into two groups: 152 (7.3%) in the FS group, with a mean age of 28 ± 15 months, and 1301 (62.7%) in the control group, with a mean age of 38 ± 16 months. Table 1 presents a comparison of variables between the FS group and the control group in the training set. In comparison to the control group, the FS group exhibited a higher proportion of males (p < 0.001). Furthermore, the FS group demonstrated significantly higher levels of ANC, AMC, RDW, and NLR compared to the control group (all p < 0.05). Conversely, the FS group had significantly lower levels of ALC, MCV, Hb, MCH, MCHC, PLT, and CRP compared to the control group (all p < 0.05). No significant differences in other laboratory variables were observed between the two groups (all p > 0.05).

**Table 1:** Comparison of variables in FS group and control group in the training set.

Variables	FS group (n = 152)	Control group (n = 1301)	P values
Age, months, mean (SD)	28 (15)	38 (16)	< 0.001
Gender (female), n (%)	54 (35.5)	686(52.7)	< 0.001
ANC, × 10^9^/L, mean (SD)	12.06 (4.18)	8.34 (4.37)	< 0.001
ALC, × 10^9^/L, mean (SD)	1.83 (0.97)	2.59 (1.36)	< 0.001
AMC, × 10^9^/L, mean (SD)	1.24 (0.51)	1.04 (0.46)	< 0.001
AEC, × 10^9^/L, mean (SD)	0.31 (1.22)	0.38 (1.38)	0.558
ABC, × 10^9^/L, mean (SD)	0.02 (0.02)	0.02 (0.02)	0.553
RBC, × 10^12^/L, mean (SD)	4.54 (0.36)	4.52 (0.50)	0.626
Hct, %, mean (SD)	35.47 (2.57)	35.82 (4.00)	0.147
MCV, fL, mean (SD)	76.91 (5.27)	78.97 (7.73)	0.001
Hb, g/L, mean (SD)	121.86 (9.55)	124.54 (13.63)	0.002
MCH, pg, mean (SD)	26.86 (1.62)	27.59 (2.68)	< 0.001
MCHC, g/L, mean (SD)	343.76 (12.543)	346.83 (31.75)	0.023
PLT, × 10^9^/L, mean (SD)	220.06 (53.00)	229.75 (56.44)	0.044
PCT, %, mean (SD)	0.22 (0.05)	0.22 (0.06)	0.438
MPV, fl, mean (SD))	9.82 (0.79)	9.79 (1.19)	0.711
RDW, fl, mean (SD)	36.09 (2.61)	35.23 (4.76)	0.001
PDW, fl, mean (SD)	10.84 (1.71)	10.72 (1.94)	0.432
CRP, mg/L, mean (SD)	8.29 (6.17)	13.35 (15.21)	< 0.001
NLR, mean (SD)	8.47 (5.15)	4.36 (3.37)	< 0.001

### Selection of predictors using the LASSO logistic regression model

**Figure 2:**
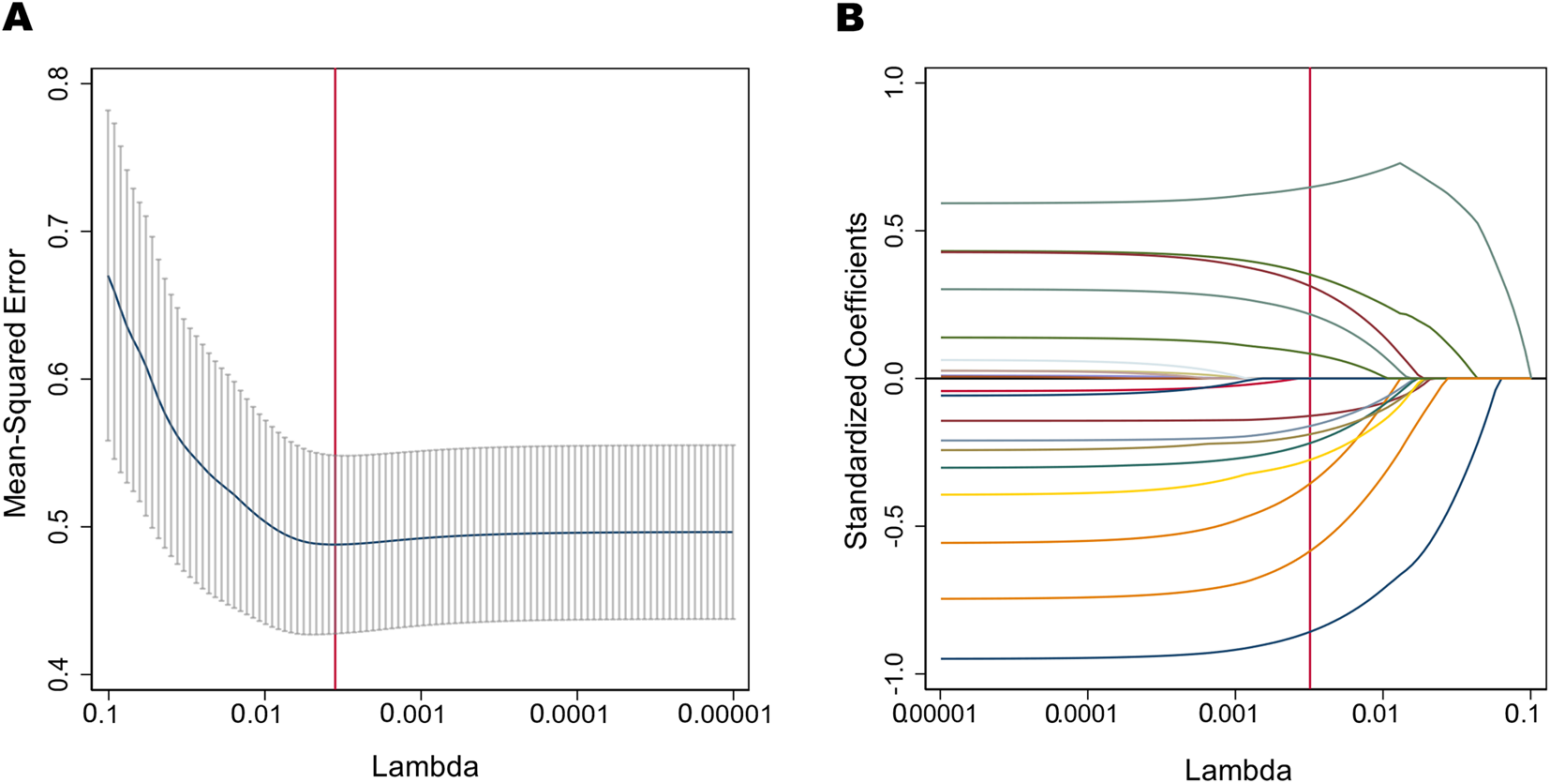
Selection of predictors using the LASSO logistic regression model. (**A**) Optimal parameter (lambda) selection in the LASSO model used fivefold cross-validation via minimum criteria. The mean-squared error curve was plotted versus lambda. Dotted vertical lines were drawn at the optimal values by using the minimum criteria and the 1 SE of the minimum criteria (the 1-SE criteria). (**B**) LASSO coefficient profiles of the 20 features. *LASSO* least absolute shrinkage and selection operator, *SE* standard error.

Since we have many covariates, we used LASSO regression for variable selection to simplify the model. Optimal parameter (lambda) selection in the LASSO model used fivefold cross-validation via minimum criteria, optimal lambda (λ = 0.0032). Do LASSO regression based on this lambda. The optimal lambda resulted in 13 variables with non-zero coefficients. These variables were Age, Gender, ANC, ALC, AMC, MCV, Hb, MCH, PLT, RDW, PDW, CRP, NLR (Fig. 2).

### Logistic regression and nomogram

**Figure 3:**
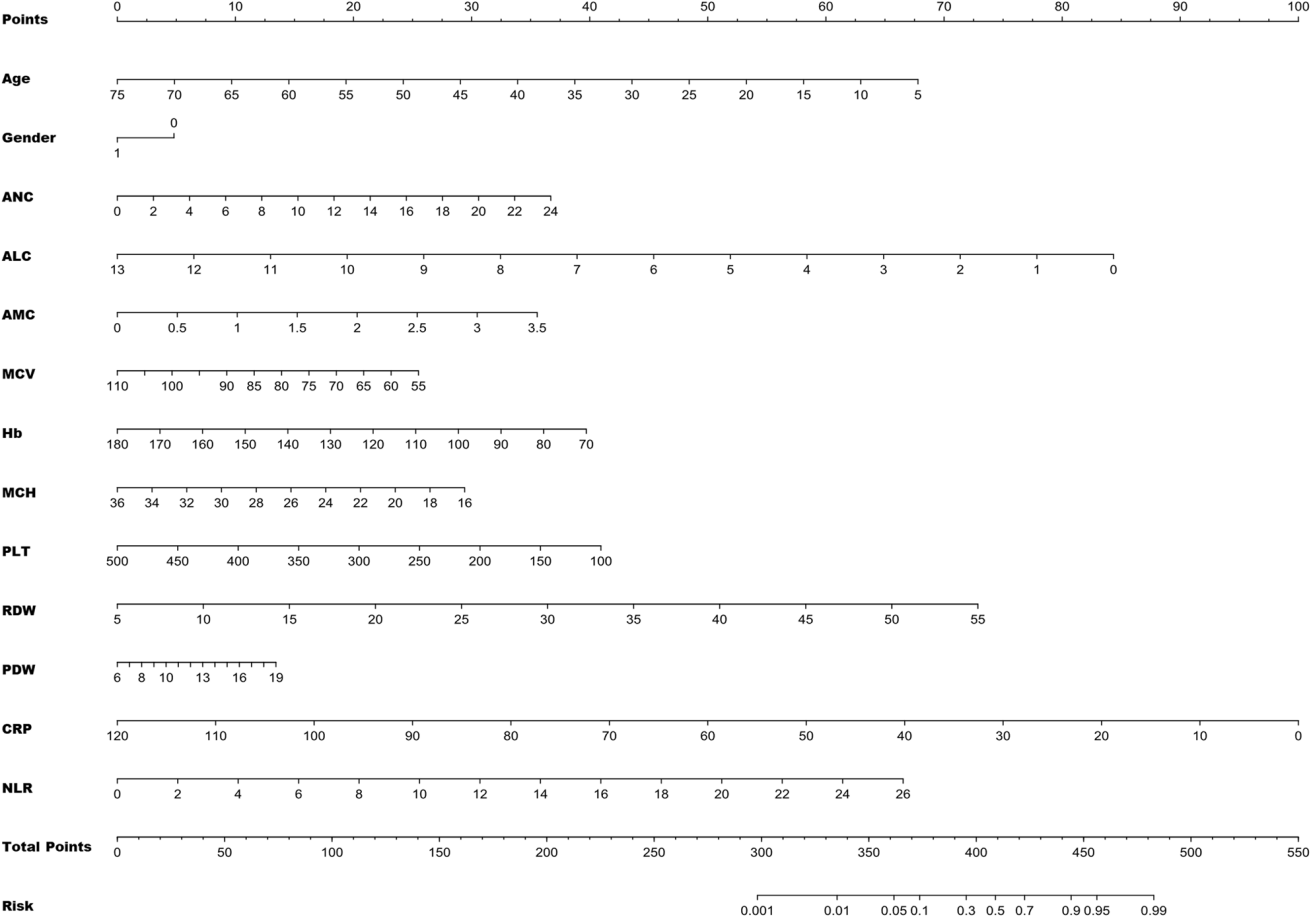
FS risk assessment tool by nomogram. "Points" correspond to the risk value of a single variable; "Total Points" is the sum of the risk values of all variables; “Risk” is determined by “Total points”.

The 13 variables selected through LASSO regression entered the logistic regression model. The expression was: logit (p) = 3.707 − 0.060*Age – 0.299*Gender + 0.095*ANC – 0.404*ALC + 0.633*AMC – 0.029*MCV – 0.022*Hb −0.092*MCH – 0.006*PLT + 0.091*RDW + 0.064*PDW – 0.052*CRP + 0.159*NLR. Among these variables, ANC, AMC, RDW, and NLR demonstrated significant positive effects on FS, while age, ALC, Hb, MCH, PLT, and CRP exhibited negative effects on FS (p < 0.05). The effects of gender, MCV, and PDW were not significant (p > 0.05). Based on the coefficients obtained from the logistic regression model, which identified risk factors for FS, a novel nomogram was developed to predict the risk of FS (Fig. 3). In the nomogram, "Points" correspond to the risk value of a single variable, which was then summed up to calculate the "Total Points". The "FS Risk" was determined based on the "Total Points". For instance, a boy with 55 months (points 20), ANC of 10 × 10^9^/L (points 15), ALC of 5 × 10^9^/L (points 50), AMC of 2 × 10^9^/L (points 20), MCV of 70 fL (points 20), Hb of 100 g/L (points 30), MCH of 30 pg (points 10), PLT of 200 × 10^9^/L (points 30), RDW of 40 fl (points 50), CRP of 50 mg/L (points 60), and NLR of 2 (points 5), would have a total of 315 points, corresponding to a risk of FS of less than 1%.

### Predictive model validation

**Figure 4:**
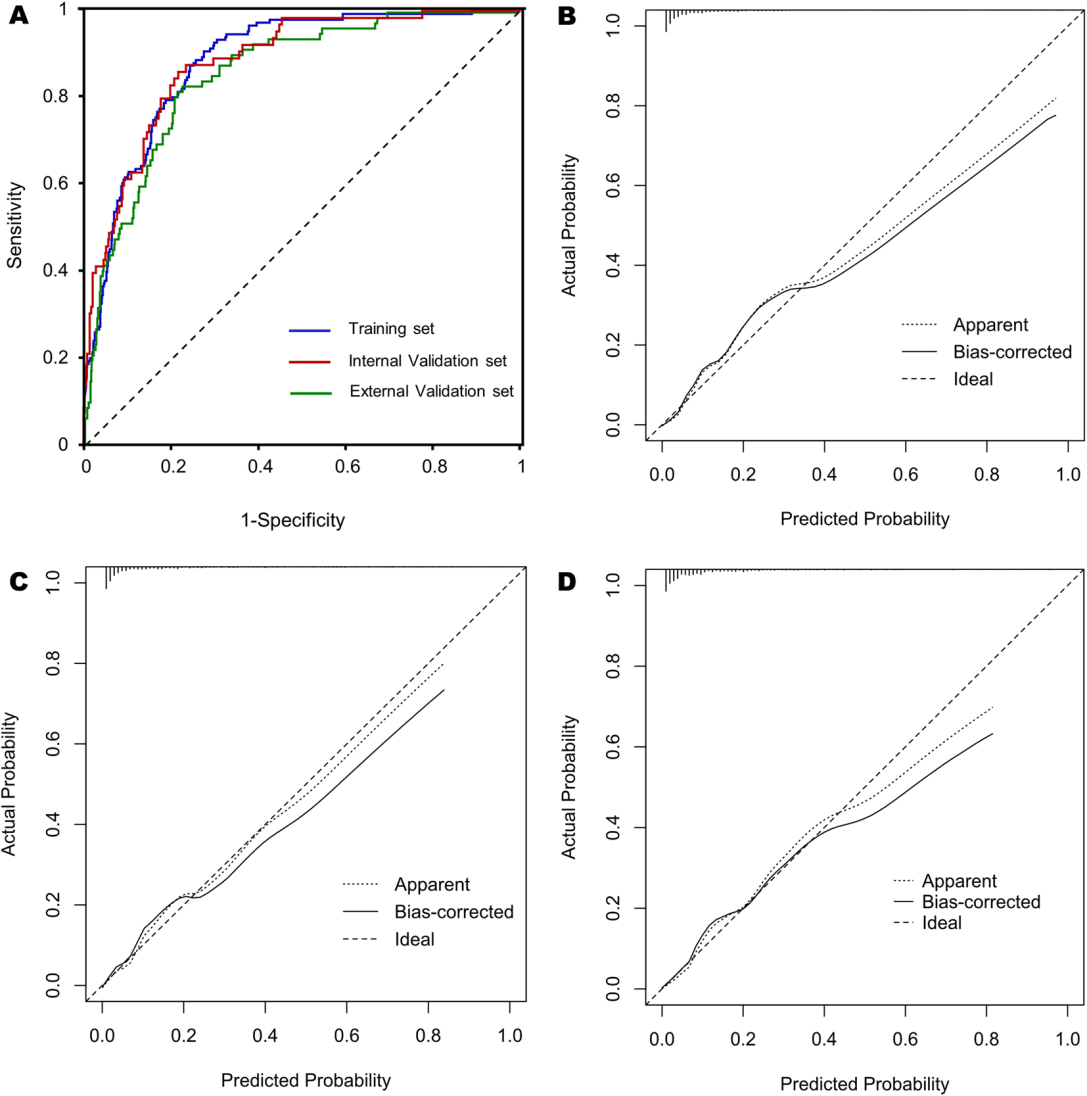
(**A**) ROC analysis. AUC = 0.884 (95% CI 0.861 to 0.908, p < 0.001) in training set; AUC = 0.883 (95% CI 0.844 to 0.922, p < 0.001) in internal validation set; AUC = 0.858 (95% CI 0.820 to 0.896, p < 0.001) in external validation set. Calibration curves of the nomogram to predict the probability of FS in training set (**B**), internal validation set (**C**), and external validation set (**D**). The “Ideal” represents the ideal reference line, the “Apparent” represents the model calibration curve, and the “Bias-corrected” represents the 1000 Bootstrap results.

The ROC curves of training set, internal validation set, and external validation set were presented in Fig. 4.A. In the training set, the AUC was 0.884 (95% CI 0.861 to 0.908, p < 0.001), with a sensitivity of 0.908 and a specificity of 0.725. In the internal validation set, the AUC was 0.883 (95% CI 0.844 to 0.922, p < 0.001), with a sensitivity of 0.862 and a specificity of 0.783. In the external validation set, the AUC was 0.858 (95% CI 0.820 to 0.896, p < 0.001), with a sensitivity of 0.829 and a specificity of 0.774. The Sensitivity, Specificity, PPV, NPV, LR+ and LR− at four suggested thresholds (100%, 95%, 90% and 85%) were summarized in Table 2.

**Table 2:** Diagnostic performance of the predictive model in the validation set.

Dataset	Cut-off score (target sensitivity)	Sensitivity	Specificity	PPV	NPV	LR +	LR-
Internal validation set	> 0.004 (100%)	1.000 (1.000–1.000)	0.115 (0.090–0.142)	0.116 (0.113–0.119)	1.000 (1.000–1.000)	1.130 (1.098–1.165)	0.000 (0.000–0.000)
	> 0.05 (95%)	0.923 (0.846–0.985)	0.634 (0.595–0.674)	0.227 (0.205–0.251)	0.986 (0.973–0.997)	2.525 (2.431–2.594)	0.121 (0.026–0.228)
	> 0.08 (90%)	0.877 (0.800–0.954)	0.728 (0.690–0.765)	0.273 (0.242–0.307)	0.981 (0.968–0.993)	3.219 (3.077–3.342)	0.169 (0.067–0.281)
	> 0.10 (85%)	0.862 (0.769–0.938)	0.776 (0.742–0.810)	0.309 (0.272–0.350)	0.980 (0.967–0.991)	3.846 (3.611–4.049)	0.178 (0.083–0.285)
External validation set	> 0.004 (100%)	1.000 (1.000–1.000)	0.088 (0.068–0.109)	0.114 (0.112–0.117)	1.000 (1.000–1.000)	1.096 (1.072–1.123)	0.000 (0.000–0.000)
	> 0.05 (95%)	0.939 (0.878–0.988)	0.555 (0.517–0.592)	0.199 (0.184–0.215)	0.987 (0.975–0.997)	2.108 (2.046–2.152)	0.110 (0.024–0.206)
	> 0.08 (90%)	0.854 (0.780–0.927)	0.691 (0.657–0.726)	0.246 (0.220–0.273)	0.976 (0.963–0.988)	2.763 (2.699–2.844)	0.212 (0.111–0.319)
	> 0.10 (85%)	0.829 (0.744–0.902)	0.763 (0.731–0.795)	0.292 (0.259–0.329)	0.974 (0.962–0.986)	3.498 (3.359–3.621)	0.224 (0.133–0.322)

The data are value (95% CI). Cut-off scores are generated from the ROC analysis of the training set.

*PPV* positive predictive value, *NPV* negative predictive value, *LR+* positive likelihood ratio, *LR−* negative likelihood ratio.

Calibration measurements was performed with C-statistic and calibration plot. In the training set (Fig. 4B), C-statistic was 0.884 (95% CI 0.861 to 0.908); calibration intercept (calibration-in-the-large) was 0.000 (95% CI −0.197 to 0.197); calibration slope was 1.000 (95% CI 0.852 to 1.148). In the internal validation set (Fig. 4C), C-statistic was 0.883 (95% CI 0.844 to 0.922); calibration intercept was 0.000 (95% CI −0.306 to 0.306); calibration slope was 1.000 (95% CI 0.779 to 1.221). In the external validation set (Fig. 4D), C-statistic was 0.858 (95% CI 0.820 to 0.896); calibration intercept was 0.000 (95% CI −0.261 to 0.261); calibration slope was 1.000 (95% CI 0.791 to 1.209). The predictive model, the internal validation set, and the external validation set showed a very good degree of fit from the calibration curves.

### Decision curve analysis and clinical impact curve

**Figure 5:**
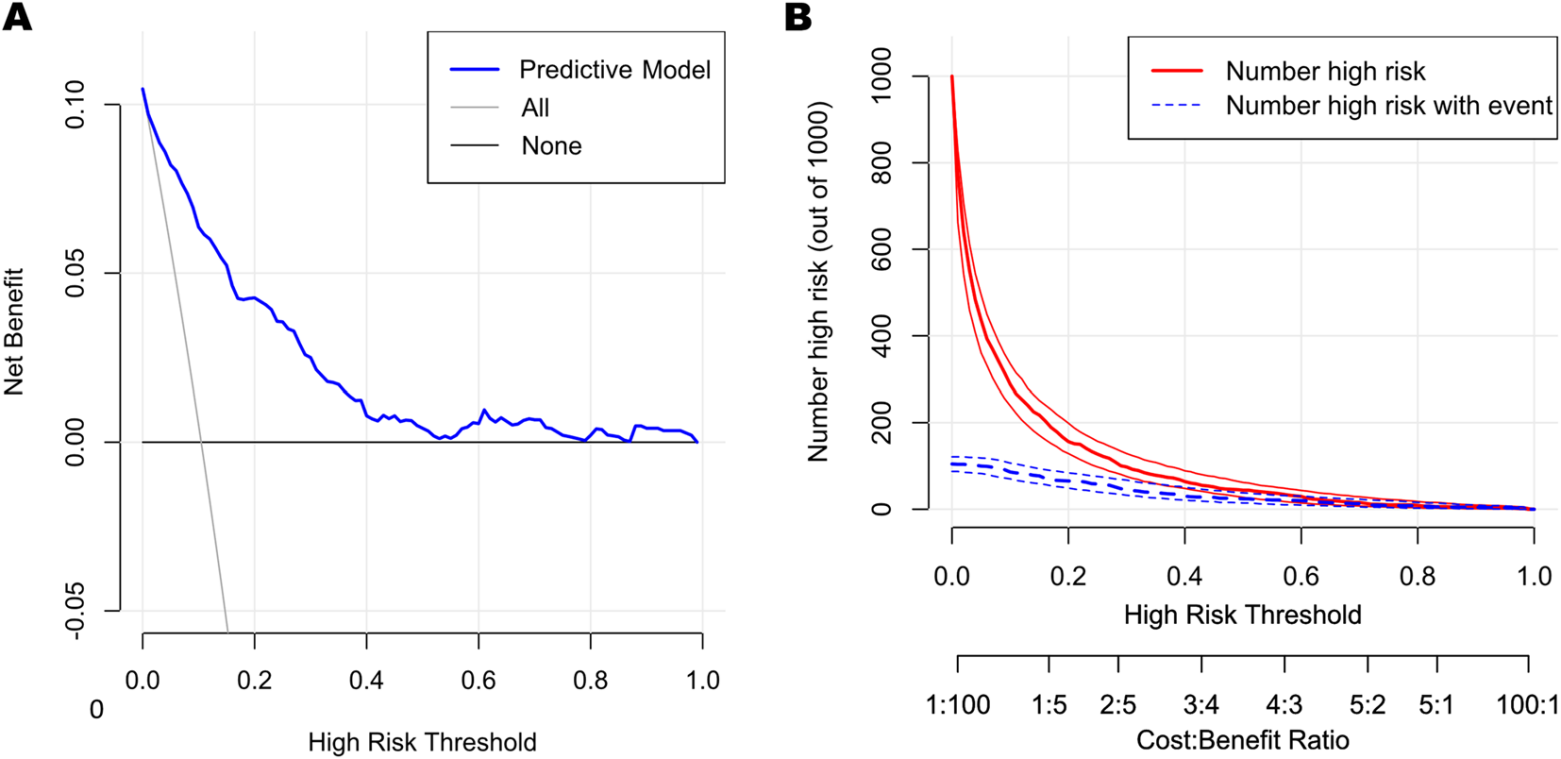
(**A**) Decision curve analysis (DCA) of the predictive model for FS. X-axis and Y-axis represent threshold probability and net benefit. The most favorable threshold probabilities for predicting FS are 0.1–0.4. (**B**) Clinical impact curve of the predictive model for FS. The red line represents the number of people judged as high risk by the model at different probability thresholds; The blue line represents the number of true positives judged as high risk by the model at different probability thresholds. The cost benefit ratio represents the proportion of cost and benefit at different probability thresholds. The Y-axis is measured in units of 1000 people.

DCA is an advanced method used to analyze the net clinical benefits of predictive models. In this study, we evaluated the clinical applicability of established FS nomograms through DCA (Fig. 5A). The results showed that the most favorable threshold probabilities for predicting FS in the training set with the nomogram were 0.1–0.4. As demonstrated by the favorable threshold probability, it indicated that the nomogram had a satisfactory clinical benefit and can assist clinicians to predict FS accurately. Figure 5B showed the CIC of the predict model and indicated that as the predicted probability increases, the population predicted by the model to have a high risk becomes increasingly consistent with the population of individuals who actually experience the outcome event.

## Discussion

Predictive models are currently receiving increasing attention in various clinical fields, including malignant tumors, sepsis, and Kawasaki disease, with a growing number of publications in these areas^19–22^. However, existing predictive models for FS demonstrate limited predictive ability^12–15^. Consequently, our study aims to explore the predictive value of CBCs in FS and construct a highly effective predictive model based on these indicators. In this retrospective analysis, a multivariable logistic regression model was constructed to predict FS. The model's results were visually presented and interpreted using a nomogram, and the predictive performance was assessed using ROC and DCA. Key findings of the study were as follows: (1) The multivariable logistic regression analysis identified four variables, ANC, AMC, RDW, and NLR, that positively influenced the likelihood of FS. Conversely, six variables including age, ALC, Hb, MCH, PLT, and CRP had a negative impact on FS. (2) The nomogram results highlighted the significant contributions of the CRP, ALC and RDW. C-statistic, a measure of discrimination and calibration, was calculated to be 0.884, indicating good predictive accuracy. (3) In the training set, the AUC was 0.884 ((95% CI 0.861 to 0.908, p < 0.001), sensitivity and specificity were 0.908 and 0.725, respectively. In the internal validation set, the AUC was 0.883 (95% CI 0.844 to 0.922, p < 0.001), sensitivity and specificity were 0.862 and 0.783, respectively. In the external validation set, the AUC was 0.858 (95% CI 0.820 to 0.896, p < 0.001), sensitivity and specificity were 0.829 and 0.774, respectively. These findings suggested that the developed predictive model exhibits favorable predictive value.

Our study revealed that ANC, AMC, RDW, and NLR had a positive influence on FS, aligning with the findings of Liu et al., who also observed significantly elevated levels of ANC and NLR in the FS group compared to the control group^15^. Similarly, other studies such as Aziz et al. and Sharawat et al. demonstrated higher RDW levels in the FS group and lower Hb and MCH levels compared to the non-seizure group^23,24^. However, it's worth noting that Liu et al. did not find a statistically significant difference in AMC levels between the FS and control groups^15^, which diverges from our study. This discrepancy may be attributed to the use of univariate analysis in their study, whereas our study employed multivariable logistic regression analysis for a more comprehensive assessment. Furthermore, our study identified age, ALC, Hb, MCH, PLT, and CRP as negative influencers of FS. Previous studies have shown that FS were most common in children between the ages of 6 months and 3 years, with a peak incidence at around 18 months^3^. Liu et al. observed significantly reduced levels of ALC, PLT, and CRP in the FS group compared to the control group^15^. Similarly, Gontko-Romanowska et al. found significantly lower ALC, PLT, and CRP levels in the FS group compared to the control group^25^. In summary, our study's findings regarding the impact of these variables on FS were in line with previous research.

It should be noted that CRP, ALC and RDW were the three indicators that showed the highest values in the nomogram, indicating their significant contributions to the predictive model. This highlights the importance of CRP and RDW in influencing FS. In a previous study conducted by Liu et al.^15^, the AUC value of NLR in predicting FS was 0.768, suggesting its predictive value was sufficient. However, in our study, when considering the nomogram, the contribution of NLR was relatively lower compared to age, CRP, RDW, and ALC. We speculate that this could be due to the inclusion of ANC and ALC in our predictive model, which partly replaced the contribution of NLR.

For the training set, the nomogram results showed a C-statistic of 0.884, indicating good predictive value. Additionally, the ROC analysis of the internal validation set yielded an AUC of 0.883 (95% CI 0.844 to 0.922, p < 0.001), further confirming the predictive capability of the model. These results suggest that the predictive model developed in this study is highly accurate, approaching an excellent level of prediction. External validation was also performed, and the ROC analysis of the external validation set showed that the AUC was 0.858 (95% CI 0.820 to 0.896, p < 0.001). This external validation further supports the good predictive value of the multivariate logistic regression model established in our study. Moreover, the minimal differences in AUC values among the training set, internal validation set, and external validation set indicate that high accuracy and consistent performance of our predictive model in predicting FS. Notably, the sensitivity in the training set reached 0.908, suggesting a high probability of accurately identifying individuals with FS among fever patients using this model. This highlights the significant clinical utility of the model.

On one hand, our study holds academic value as the AUC values achieved 0.884, 0.883 and 0.858 in the training set, internal validation set and external validation sets respectively, indicating the strong predictive performance of the regression model. This contributes to a deeper understanding of FS and enables clinicians to identify patients at risk of developing FS within the population, advancing FS prediction research. The high consistency in the three datasets suggests the generalizability of the predictive model, making it suitable for practical extension and application in clinical practice. On the other hand, in terms of practical significance and clinical value, our study exhibited remarkable sensitivity, indicating that the model effectively distinguishes patients who are likely to progress to FS. This highlights its potential clinical utility. Additionally, the nomogram, which visually represents complex regression equations, is location-independent and can be easily understood and utilized by healthcare professionals at all levels, as well as the public without medical backgrounds, with simple training. It enables personalized risk prediction and has gained increasing attention and utilization in medical research^20–22^. Furthermore, the demographic variables (age, gender) and blood indicators used in this study are readily accessible in hospitals of all levels, do not require advanced medical equipment, have high acceptance, and have low economic burden. This makes the regression model highly applicable in clinical settings, emphasizing its clinical value.

One of the limitations of our study was its single-center design, which lacks validation in diverse populations. It remains unclear whether our results can be generalized to different regions and races. To improve the accuracy and representativeness of the predictive model, multicenter studies with larger sample sizes are needed. While the validation sets' AUC values in this study were high, the scarcity of studies on FS predictive models calls for further research to validate our model. Moreover, the specificity of the constructed model in this study was not sufficiently high, which may limit its ability to identify febrile children without seizures. Strengthening the predictive ability and practical clinical application value of the model by achieving higher specificity and sensitivity is an avenue for future research. Another aspect for improvement in this study could be the inclusion of additional demographic variables and laboratory indicators. However, it should be noted that in most cases, the additional laboratory indicators may lead to unnecessary medical interventions, causing discomfort for children and financial burdens for families. Additionally, adding more variables may significantly increase the complexity of the model, hindering its widespread clinical application. In conclusion, further research is warranted to enhance the prediction of FS in children, with future advancements in testing techniques and algorithms.

## Data Availability

The datasets generated during and/or analyzed during the current study are available from the corresponding author on reasonable request.
