# Author Correction: Development and validation of a predictive model for febrile seizures

**DOI:** 10.1038/s41598-023-47491-0

**Published:** 2023-12-15

**Authors:** Anna Cheng, Qin Xiong, Jing Wang, Renjian Wang, Lei Shen, Guoqin Zhang, Yujuan Huang

**Affiliations:** Department of Emergency, Shanghai Children’s Hospital, School of Medicine, Shanghai Jiao Tong University, Shanghai, China

Correction to: *Scientific Reports* 10.1038/s41598-023-45911-9, published online 31 October 2023

In the original version of this Article, the affiliation of the authors was incorrectly given as ‘Children’s Hospital of Shanghai, Shanghai, China’. The correct affiliation is:

Department of Emergency, Shanghai Children’s Hospital, School of Medicine, Shanghai Jiao Tong University, Shanghai, China

The original Article has been corrected.

